# Designing Electron-Deficient Diketone Unit Based Non-Fused Ring Acceptors with Amplified Optoelectronic Features for Highly Efficient Organic Solar Cells: A DFT Study

**DOI:** 10.3390/molecules28083625

**Published:** 2023-04-21

**Authors:** Muhammad Usman Khan, Faiza Shafiq, Sanaa S. Al Abbad, Junaid Yaqoob, Riaz Hussain, Zainab H. A. Alsunaidi, Ghulam Mustafa, Shabbir Hussain

**Affiliations:** 1 Department of Chemistry, University of Okara, Okara 56300, Pakistan; 2 Department of Chemistry, University of Agriculture, Faisalabad 38000, Pakistan; 3 Department of Chemistry, College of Science, Imam Abdulrahman Bin Faisal University, Dammam 31441, Saudi Arabia; 4 Institute of Chemistry, Khwaja Fareed University of Engineering & Information Technology, Rahim Yar Khan 64200, Pakistan

**Keywords:** DFT, electron-deficient diketone units, end-capped modification, organic solar cell, optoelectronic properties

## Abstract

Organic solar cells (OSCs) made of electron-acceptor and electron-donor materials have significantly developed in the last decade, demonstrating their enormous potential in cutting-edge optoelectronic applications. Consequently, we designed seven novel non-fused ring electron acceptors (NFREAs) (BTIC-U1 to BTIC-U7) using synthesized electron-deficient diketone units and reported end-capped acceptors, a viable route for augmented optoelectronic properties. The DFT and TDDFT approaches were used to measure the power conversion efficiency (PCE), open circuit voltage (Voc), reorganization energies (λ_h_, λ_e_), fill factor (FF), light harvesting efficiency (LHE) and to evaluate the potential usage of proposed compounds in solar cell applications. The findings confirmed that the photovoltaic, photophysical, and electronic properties of the designed molecules BTIC-U1 to BTIC-U7 are superior to those of reference BTIC-R. The TDM analysis demonstrates a smooth flow of charge from the core to the acceptor groups. Charge transfer analysis of the BTIC-U1:PTB7-Th blend revealed orbital superposition and successful charge transfer from HOMO (PTB7-Th) to LUMO (BTIC-U1). The BTIC-U5 and BTIC-U7 outperformed the reference BTIC-R and other developed molecules in terms of PCE (23.29% and 21.18%), FF (0.901 and 0.894), normalized Voc (48.674 and 44.597), and Voc (1.261 eV and 1.155 eV). The proposed compounds enclose high electron and hole transfer mobilities, making them the ideal candidate for use with PTB7-Th film. As a result, future SM-OSC design should prioritize using these constructed molecules, which exhibit excellent optoelectronic properties, as superior scaffolds.

## 1. Introduction

Photovoltaic technology using organic solar cells (OSCs) is seen as auspicious future development due to their unique characteristics, which include being printable over large areas, semi-transparent, flexible, and lightweight [1,2,3,4,5]. The PCE of single-junction OSCs has surpassed 17% because of the expansion of new non-fullerene acceptors (NFAs) [6,7,8,9,10]. Fused ring acceptor-donor-acceptor (A-D-A) type assemblies are typically found in today’s most effective NFAs because of their minimal energy loss, tunable energy levels and stronglight absorption [11,12,13,14,15,16]. Usually, the large central cores, denoted by the symbol “D unit,” are fused-ladder-type building blocks. These cores encourage the p-electrons delocalization and make it easier for molecules to pack closely together [11,12,15,17]. While molecules like INIC, IDCIC, and ITOT can improve device performance, the multi-step synthesis procedures required to produce them always lead to low yields and high costs [18,19,20,21]. In light of this, NFREAs have been designed with p-conjugated backbones that are either fully or partially unfused-ladder rings to intervene in molecular planarity via intramolecular noncovalent interactions involving F/H, N/S, and O/S noncovalent bonds [22,23,24,25].

In addition to their diverse building blocks and synthetic simplicity, these NFREAs exhibit high electron mobility and broad absorption, just like their fused-core counterparts, resulting in comparable photovoltaic performances [24,26,27,28,29]. For instance, OSCs based on NFREAs with an absorption range greater than 900 nm, and an O/S noncovalent interaction that originated from nearly planar molecular geometry were reported by Huang et al. to have a PCE greater than 14.5% [29]. BDC-4F-C8, a new NFREAs system with PCE of 12.53% and energy loss of 0.51 eV, was developed by Luo et al. It consisted of a fluorinated 1,1 dicyanomethylene-3- indanone (DFIC) terminal unit, a cyclopentadithiophene flanking unit, and a 5,7-bis(2-ethylhexyl)-4H,8H-benzo [1,2-c:4,5-c0] dithiophene-4,8-dione (BDD) as a central core unit [30].

These findings suggest that NFREAs created by incorporating electronegativity atoms with intramolecular noncovalent interactions (NCI) can significantly contribute to improved photovoltaic performance. Because of their elevated HOMO energy levels, NFREAs containing non-fused electron-donating rings or alkyloxy-substituted benzene have typically been restricted to pairing with polymer donors such as PM6, PTQ10 having wide bandgap and lower-lying HOMO level. As a result, the energy profile of hole transfer from NFREAs to donors is unfavorable [31].

Thus, electron-deficient D moiety units are needed to lock the molecular geometry through intramolecular NCIs and achieve low-lying HOMO energy levels. Taking this into account, the need for electron-withdrawing properties and the desire for a noncovalent intramolecular interaction appear to be satisfied by electron-deficient diketone units. The O/S intramolecular interactions in thieno[3,4-c]pyrrole-4,6-dione (TPD) NFREAs were reported by Chen’s group [32]. TPD core exhibited additional quinoidal character than that of commonly encountered alkyloxy-substituted benzene analogues, resulting in improved coplanarity of NFREAs skeleton and larger p-electrons delocalization. When combined with wide bandgap polymer donors, electron affinity is increased by the diketone groups’ which prevents the HOMO levels from being efficiently boosted. Additionally, the molecular planarity is enhanced by the O/S intramolecular NCI, which also boosts the ICT effect. Thus, when using PBDB-T, the TPD-based device attained a PCE of 10.12%.

The 2,20-bithiophene-3,30-dicarboxyimide (BTI) unit, one of many electron-deficient diketone units, was used as the basis of many superior polymer semiconductors [33,34,35,36]. The π-electron delocalization in BTI units gives them potent electron-drawing properties. For an all-polymer solar cell, for instance, Guo’s team reported a PCE of 14.3% using an acceptor polymer holding BTI entity [37]. The presence of these characteristics suggested that the BTI unit could be used to build NFREAs for efficient OSCs. In light of the preceding, Luo et al. reported a pair of straightforward electron acceptors (BTIC-4F and TPDC-4F NFREAs) comprised of BTI and TPD electron-deficient diketone cores, correspondingly, linked to the DFIC terminals via a CPDT bridge [38].

End-capped modification is a good way to design novel OSC materials with outstanding photovoltaic properties [39,40,41,42,43]. Our work suggests that building NFREAs with end-capped modifications and electron-deficient diketone units is viable for performant and economical OSCs. We also shed light on the sophisticated molecular design behind efficient yet simple electron acceptors. Using end-capped engineering, which replaces the terminal units with new electron-withdrawing groups, we created seven new simple electron acceptors (BTIC-U1 to BTIC-U7). The photovoltaic properties of proposed compounds are predicted using DFT and TDDFT computations. This theoretical perspective will allow computational scientists to utilize this suitable approach for designing novel OSC materials and experimental scientists to synthesize these extremely effective predicted molecules for solar cell applications.

## 2. Result and Discussion

### 2.1. Chemistry of Molecules

**Figure 1:**
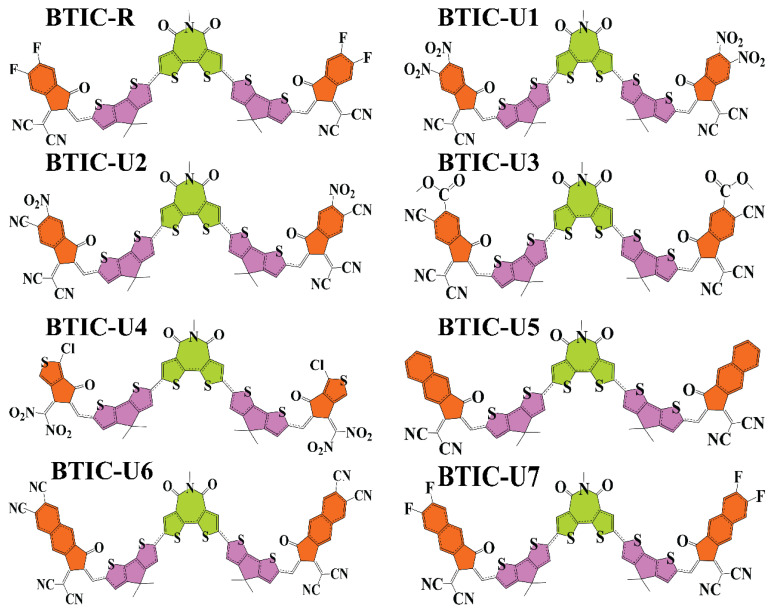
ChemDraw structures of BTIC-R and BTIC-U1 to BTIC-U7 molecules.

In this study, BTIC-R [38], which contains 5-methyl-4H-dithieno[3,2-c:2’,3’-e]azepine-4,6(5H)-dione as the central acceptor unit (A1), served as our reference molecule. 2-(5,6-difluoro-2-methylene-3-oxo-2,3-dihydro-1H-inden-1-ylidene)malononitrile, which functions as the peripheral acceptor part (A2) and is directly connected to the donor fragments 4,4-dimethyl-4H-cyclopenta[2,1-b:3,4-b’]dithiophene, makes the A2-D-A1-D-A2 type assembly of overall molecule. The BTIC-R end sites were subjected to a series of modifications, and the development of seven brand-new molecules (BTIC-U1 to BTIC-U7) was carried out. Consequently, new electron acceptor groups were substituted for the formally stated A2. These new electron acceptor units are as follows: 2-(2-methyl-5,6-dinitro-3-oxo-2,3-dihydro-1H-inden-1-ylidene)malononitrile (BTIC-U1), 2-(6-cyano-2-methylene-5-nitro-3-oxo-2,3-dihydro-1H-inden-1-ylid(dinitromethylene)-5-methylene-1,3,5,6-tetrahydro-4H-cyclopenta[c]thiophen-4-one (BTIC-U4), (Z)-2-(2-ethylidene-3-oxo-2,3-dihydro-1H-cyclopenta 2-(6,7-difluoro-2-methylene-3-oxo-2,3-dihydro-1H-cyclopenta[b]naphthalen-1-ylidene)malononitrile (BTIC-U5), 1-(dicyanomethylene)-2-methylene-3-oxo-2,3-dihydro-1H-cyclopenta[b]naphthalene-6,7-dicarbonitrile (BTIC- (BTIC-U7). Figure 1 presents the ChemDraw assemblies of BTIC-R and BTIC-U1 to BTIC-U7 molecules discussed earlier in this paragraph.

### 2.2. Method Selection and Optimized Geometries

**Figure 2:**
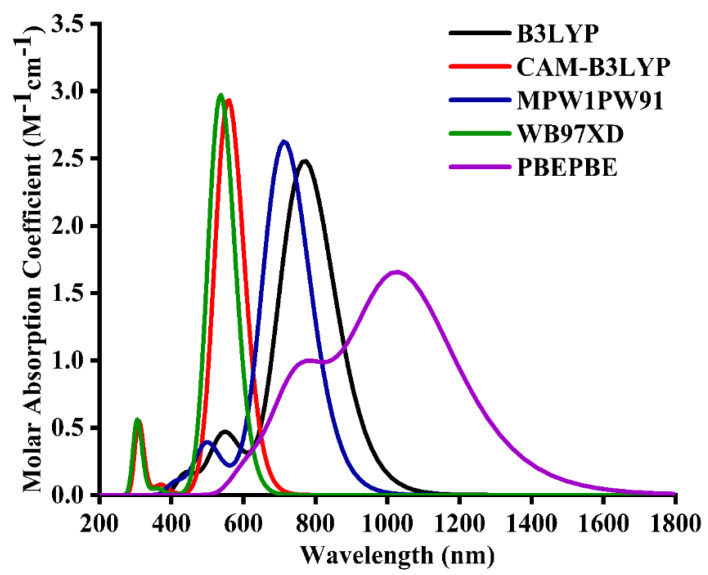
Comparison of BTIC−R solvent phase absorption values at diverse functionals.

After the geometrical optimization of BTIC-R, its time-dependent-DFT calculations were made in the excited state, specifically the cited chloroform solvent. Chloroform was used as the solvent of choice due to its implementation on the reference molecule in the cited literature. Utilizing five distinct functionals (PBEPBE, ωB97XD, MPW1PW91, CAM-B3LYP, B3LYP) and a basis set of 6-31G*, the BTIC-R molecule’s absorption measurements were analyzed. The reason for using these functional is to investigate the functional that could lead to desirable results in terms of *λ_max_*. Figure 2 demonstrates that the *λ_max_* level of the BTIC-R molecule for these functionals is 769 nm, followed by 558 nm, 713 nm, 537 nm, and 1034 nm in that order. The *λ_max_* of the BTIC-R molecule was measured experimentally to be 696 nm [38] (see Figure 2), and DFT calculations based on *λ_max_* values suggest that the MPW1PW91/6-31G* has the highest concordance with the experimentally obtained *λ_max_*. Therefore, this functional was selected to carry out the research’s auxiliary theoretical calculations. As a result, the functional and basis set were applied to optimize BTIC-U1 to BTIC-U7 molecules. It’s common knowledge that a molecule’s structure significantly affects its optoelectronic properties. Delocalization of π-electrons allows for a more rapid and efficient charge transfer between BTIC-R and BTIC-U1 to BTIC-U7 molecules, resulting in a longer conjugation.

### 2.3. Quantum Mechanical Descriptors

Electronic properties, absorption and charge transmission of investigated molecules are profoundly affected by the energies of frontier molecular orbitals (LUMO and HOMO) [44,45,46,47]. The valence band, or HOMO, is where electrons are given away, while the conduction band, or LUMO, is where they are accepted [48,49]. The energy gap (*E*_gap_) is a standard identifier of photovoltaic (PV) and solar cell (SC) devices; it is a measure of how much energy is needed to split an atom’s electrons apart [50]. Band gaps in organic solar cells can be narrower to increase efficiency [51]. Efficient and reliable PV devices require molecules with the smallest possible band gap value. Band gaps (*E*_gap_), HOMO, and LUMO energies for BTIC-R and BTIC-U1–U7 are shown in Table 1 below.

**Figure 3:**
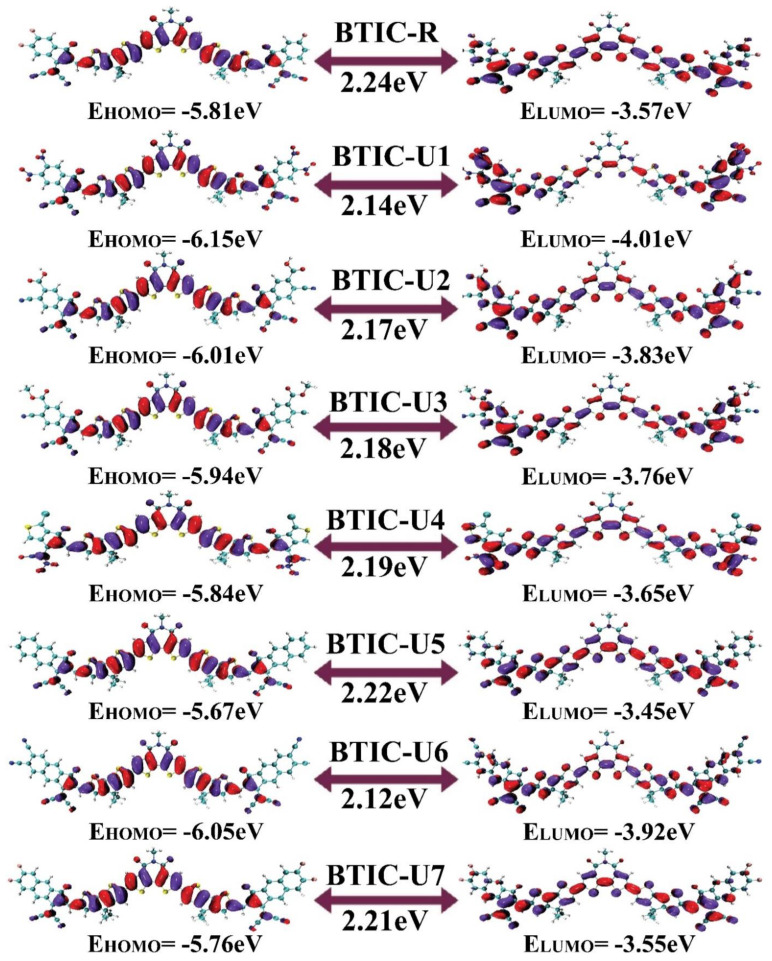
Depiction of FMOs distribution for BTIC−R and BTIC−U1 to BTIC−U7.

In Figure 3, HOMO-LUMO plots for every molecule (built from their optimized geometries) and their corresponding *E*_gap_ values are portrayed. The ground state’s (HOMO) density of charges is centralized on the molecule’s D and A1 parts, demonstrating that charge can be efficiently transferred from the ground to excited states. In the excited state, electron density also moves towards end-group acceptors (LUMO). In addition, in both FMOs under consideration, the molecule’s predominantly planar topology is indicated by the fact that the charge density is spread out over nearly the entire compound.

The HOMO energy of the BTIC-R molecule was measured to be −5.81 eV, and the LUMO energy of the same molecule was estimated to be −3.57 eV. This molecule also displayed an *E*_gap_ of 2.24 eV, which is higher than all proposed compounds. The HOMO of the BTIC-U1 molecule was shown to have the lowest energy of all the molecules studied, which indicates that the HOMO of the BTIC-U1 molecule is the most stable of all the molecules. Nitro groups are present in the end-capped acceptor groups of BTIC-U1 molecules; these groups have the potential to contribute to the molecules’ stability.

In addition, The BTIC-U6 molecule possesses the HOMO with the second highest degree of stability, right behind the BTIC-U1 molecule. This is because the HOMO of BTIC-U6 molecule is lesser than any other molecule, coming in at −6.05 eV, and it also has a significantly higher amount of cyano units than any other molecule. In general, the order of decreasing HOMO energy levels across all investigated molecules is BTIC-U5 > BTIC-U7 > BTIC-R > BTIC-U4 > BTIC-U3 > BTIC-U2 > BTIC-U6 > BTIC-U1, and the declining array of the LUMOs for BTIC-R and BTIC-U1 to BTIC-U7 molecules is as follows: BTIC-U5 > BTIC-U7 > BTIC-R > BTIC-U4 > BTIC-U3 > BTIC-U2 > BTIC-U6 > BTIC-U1. Therefore, in both of the evaluated FMOs, the energy levels present in BTIC-U1 and BTIC-U6, respectively, are incredibly low and rank second to last. Moving on, the *E*_gap_ in the BTIC-U6 molecule is 2.12 eV, which is less than what is found in the BTIC-R molecule and every other designed molecule. Again, the BTIC-U1 molecule had the second smallest *E*_gap_ of all the molecules tested (2.14 eV). In addition, all newly created molecules have an *E*_gap_ lower than the *E*_gap_ of the BTIC-R molecules. This demonstrates that the modified molecules are pretty capable of transferring charge effectively. According to the results obtained from Table 1, the decreasing order of all molecules that were studied concerning the *E*_gap_ is BTIC-R > BTIC-U5 > BTIC-U7 > BTIC-U4 > BTIC-U3 > BTIC-U2 > BTIC-U1 > BTIC-U6.

### 2.4. Absorption Spectrum

The spectral analysis of BTIC-R and BTIC-U1 to BTIC-U7 molecules was performed using the previously determined MPW1PW91/6-31G*. Table 2 and Table 3 present the assessed statistics for the solvent (chloroform) and gaseous phases, respectively.

**Figure 4:**
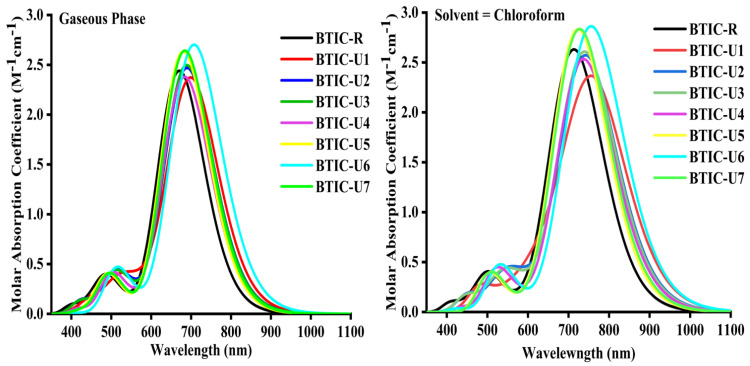
Graphical demonstration of UV–Vis absorption of BTIC−R and BTIC−U1 to BTIC−U7 in Chloroform solvent (**right**) and gaseous phase (**left**).

As seen in Figure 4, the absorbance spectrum of each of the compounds that are the subject of this investigation spans the 350 nm to 1100 nm interval in both tested phases. The maximum wavelength (*λ_max_*) of the BTIC-R molecule is 670 nm when it is in the gaseous phase, but it is 713 nm in chloroform (CHCl_3_). Molecules in the BTIC-U1 to BTIC-U7 series have *λ_max_* values between 680 and 707 nm in the gaseous phase and between 723 and 761 nm in the CHCl_3_ solvent. Compared to the BTIC-R molecule, all modified molecules exhibited a bathochromic shift in their *λ_max_* in solvent and gaseous phases, as shown by this UV-visible spectral analysis. And this bathochromic shift displayed by BTIC-U1 to BTIC-U7 molecules is between 18–37 nm in the gas and 10–48 nm in the solvent phase concerning the BTIC-R molecule.

In both the gas phase and the CHCl_3_, the increasing *λ_max_* sequence of BTIC-R and BTIC-U1 to BTIC-U7 molecules is as follows: BTIC-R < BTIC-U5 < BTIC-U4 < BTIC-U7 < BTIC-U3 < BTIC-U2 < BTIC-U1 < BTIC-U6 and BTIC-R < BTIC-U5 < BTIC-U7 < BTIC-U4 < BTIC-U3 < BTIC-U2 < BTIC-U6 < BTIC-U1, sequentially. All studied compounds in chloroform solvent exhibited a bathochromic shift relative to the gas phase due to the stabilisation of the polar excited state by a polar solvent. The shorter *E_gap_* of all the molecules resulted in a longer wavelength as a result of the inverse correlation between wavelength and energy.

The findings indicate that BTIC-U1 has a *λ_max_* of 761 nm in a solvent medium, pointing to a notable end-capped acceptor that may have affected its *λ_max_*. The oscillator strength (f) is a dimensionless factor that determines the photovoltaic cell’s optical properties and the calculation of the strength of the radiation generated upon the electrical excitation of two energy levels. The excitation energy of OSC is also crucial to enhancing their photovoltaic or optoelectronic properties. All the molecules we’ve designed have low excitation energies relative to the standard BTIC-R molecule in the solvent phase, making them ideal for photovoltaic or optoelectronic applications.

Table 2 and Table 3 show data from the gas and solvent phases, highlighting significant HOMO→LUMO (≤95%) transitions or assignments for various molecules. These results apply to all BTIC-U1 to BTIC-U7 designed molecules and the BTIC-R reference molecules. This quality is also present in molecules engineered to have superior optoelectronic properties. Figure 4 depicts the simulated absorption spectra of molecules BTIC-U1 through BTIC-U7, and the reference molecule BTIC-R attained in chloroform and gas phases, illustrating each molecule’s broader and more intense peak. The tabulated results in Table 2 and Table 3 show that the *λ_max_* values for BTIC-U1 to BTIC-U7 are significantly impacted by the end-capped unit modifications, resulting in a 92% HOMO-LUMO transition, low excitation energies and a red shift in the UV/Visible spectrum. Thus, all of these molecules (BTIC-U1 to BTIC-U7) are superior to BTIC-R, the standard molecule, in their optoelectronic properties. Improving the photovoltaic capabilities and adjusting the photophysical characteristics of proposed compounds is easily accomplished through end-capped unit modification. That’s why the newly designed molecules BTIC-U1 through BTIC-U7 have better optoelectronic properties than BTIC-R.

### 2.5. Light Harvesting Efficiency (LHE)

**Figure 5:**
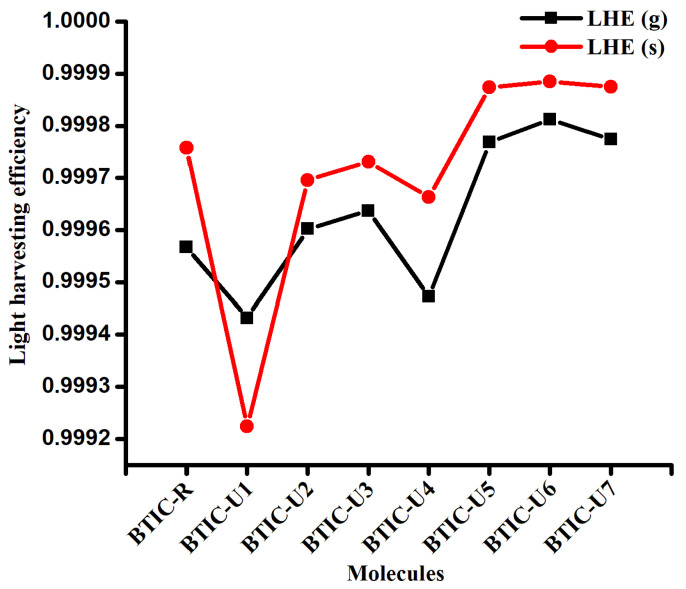
Light harvesting efficacy for BTIC-R to BTIC-U7 molecules in gas phase and in solvent.

Every component that goes into the SCs needs to generate a charge following the light collection process, also referred to as the LHE. This LHE affects the device’s solar efficiency, which in turn is affected by the oscillator strength due to its effect on short-circuit current generation. The LHE was determined for each of the compounds that were investigated by applying the Equation (1).

(1) $$\eta_{\lambda }=1-10^{-f}$$

where the LHE is denoted by $\eta_{\lambda }$ and *f* denotes oscillator strength. In the following Table 4, the LHE values that were computed for molecules of BTIC-R and BTIC-U1 to BTIC-U7 in both the gas phase and the chloroform solvent are listed. The BTIC-U5 to BTIC-U7 molecules in the solvent phase displayed higher LHE values than BTIC-R due to the more significant oscillator strengths possessed by these molecules (Figure 5). Because of the greater oscillation strength of BTIC-U6, LHE is at its highest level there. The findings indicate that the LHE of these small molecules is significantly affected by the terminal acceptor moieties.

### 2.6. Dipole Moment

There are few properties more crucial than crystallinity and solubility, and both are profoundly affected by dipole moment (μ). These characteristics play an essential role in establishing polarization situation in the required solvent for efficient OSCs compounds. Continuous charge transfer is enabled by the excellent polar solubility, superior crystallinity and tight molecular assembling of molecules with planar and ordered geometries containing significant dipole moments. A molecule’s solubility and crystallinity in polar substances increase with increasing dipole moment, which improves charge transmission. In general, molecules with zero dipole moments cannot be dissolved by organic polar solvents like chloroform.

**Figure 6:**
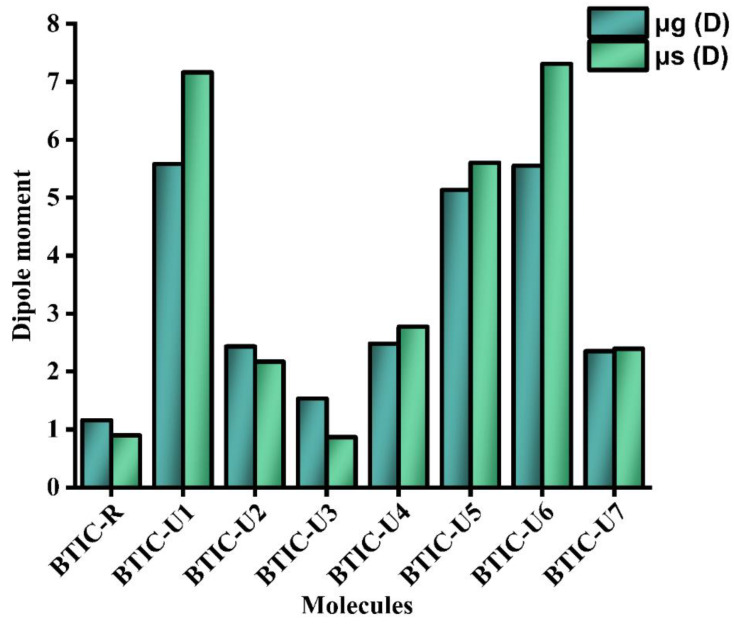
Graphical illustration of Dipole moment in the gas phase and solution.

However, this relationship is not constant because the charge transfers properties and solubility of each molecule depend on its specific molecular arrangement. Both the solvent and gaseous states are represented in Table 5 and Figure 6 by the μ values of the molecules BTIC-R and BTIC-U1 to BTIC-U7. In the solvent state, the order is, i.e., BTIC-U3 < BTIC-R < BTIC-U2 < BTIC-U7 < BTIC-U4 < BTIC-U5 < BTIC-U1 < BTIC-U6. The μ of the BTIC-U3 molecule was lower than the BTIC-R molecule in the solvent phase. However, it was greater than BTIC-R for the other molecules. Regarding solubility and polarity, BTIC-U6 had a huge dipole moment, possibly due to the cyano groups at its ends.

Compared to the reference molecule, the incremental exchange of the μ was found in the range of 1.532–5.582 Debye (when measured in gas) and 0.866–7.307 Debye (when measured in solvent), as shown in Table 5. These findings validate the simulated materials’ applicability for organic solar cells by proving their claim that crystalline behavior becomes more stable in the solvent. In-depth comparisons suggest that BTIC-U6, with its maximum μ value of 7.307 Debye, is the more crystalline material. An in-depth view of comparative analysis prompts BTIC-U6 as a more crystalline material because of its highest dipole moment values of 7.307 Debye. Additionally, all modeled entities have changed to have more dipole moments, guaranteeing accurate modeling for highly efficient polymer solar cells.

### 2.7. Density of State

**Figure 7:**
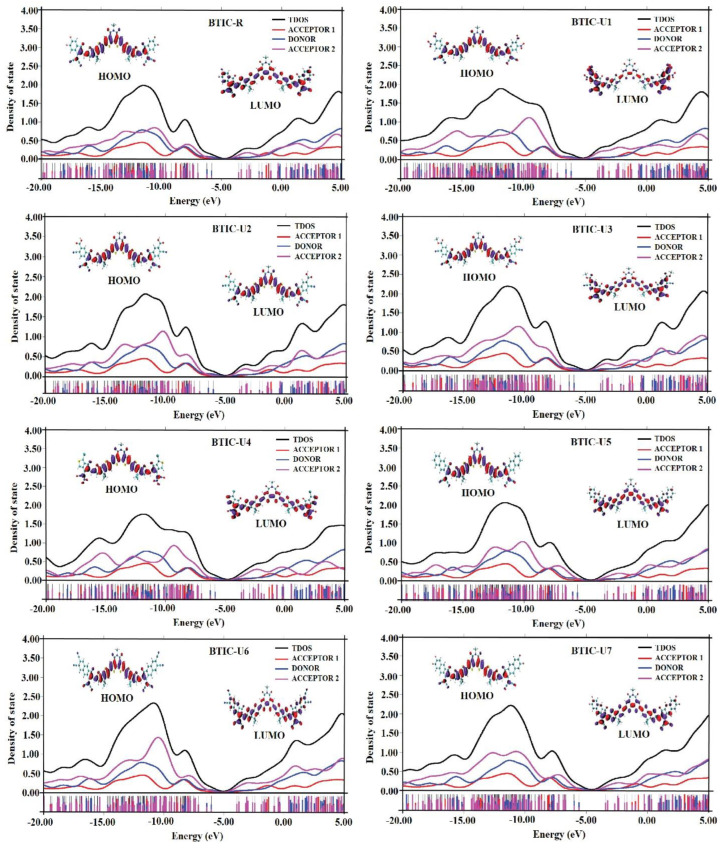
DOS plots of BTIC−R and BTIC−U1 to BTIC−U7.

Studies of DOS are necessary for understanding how the total and partial DOS in the charge mobility of molecules validate the utilities of individual units (acceptor1, donor, and acceptor2) and the inclusive performance of investigated molecules. When analyzing the FMOs regarding the Mulliken charge density, it is essential to determine the order in which the FMOs are arranged. Calculations of the DOS for each of the molecules under investigation were carried out by employing the MPW1PW91/6-31G* methodology. The DOS plots have an x-axis that shows the total amount of energy and a y-axis that shows the relative intensity of that value. For this investigation, studied compounds are cut up into three different units—acceptor1, donor, and acceptor2—so that the researchers could examine the contributions made by each fragment to the FMOs. In Figure 7, black, blue, pink, and red lines represent the total contribution of the molecules (BTIC-R and BTIC-U1 to BTIC-U7) as a whole, contributions of the donor moieties, acceptor2 and acceptor1, respectively.

In addition, the numerical contribution that each distinct sector of BTIC-R and BTIC-U1 makes to BTIC-U7 molecules is mentioned in Table 6. The donor group is an essential factor in the overall elevation of the ground state for all of the recently developed molecules and the BTIC-R molecule (HOMO). The contributions of both acceptor2 and acceptor1 to the HOMO energy level are similar but modest. However, acceptor1 contributes slightly more to the HOMO energy level than acceptor2.

Acceptor2 is responsible for most of the excited state’s (LUMO) contribution. Based on these findings, it can be deduced that charges will be transferred via subsequent conjugation from the ground state of the donor fragment (electron-rich) part of molecules to the A2 unit in their excited state. Moreover, these results are consistent with the FMO analysis of BTIC-R and BTIC-U1 to BTIC-7, as shown in Figure 7.

### 2.8. Electrostatic Potential (ESP)

ESP shows the distribution of charges throughout a molecule in three dimensions and draws attention to the various locations within the molecule where electrons can be found. An ESP analysis was carried out on the molecules under consideration, in order to make a prediction regarding the reactive potential of molecular frameworks.

**Figure 8:**
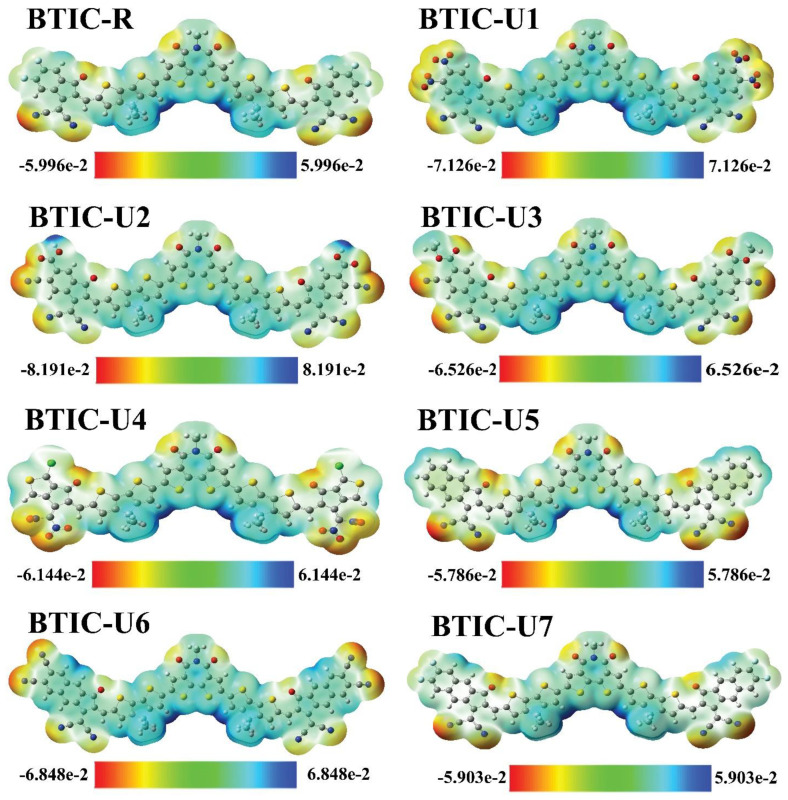
ESP maps of BTIC−R and BTIC−U1 to BTIC−U7.

ESP maps depict the three-dimensional distribution of electronegative substances, lone pairs and electrons readily available for nucleophilic action. Red indicates an area with a high concentration of electrons on the ESP maps. Green represents a neutral region, and blue represents an area with a low concentration of electrons. Figure 8 displays the BTIC-R and BTIC-U1-BTIC-U7 ESP color maps.

Because of the high electron density above these positions, ESP maps show the N and O atoms in the outer acceptor molecular regions as deep red. Likewise, the oxygen atoms in the core’s acceptor region show up in red, making the existence of electron density in those regions obvious. Donor parts with methyl groups and thiophene rings are shown to have severe electron depletion in these ESP maps, as indicated by their bluish coloration.

### 2.9. Analysis of Charge Mobility

By computing the RE of BTIC-U1 to BTIC-U7 and BTIC-R molecules at the MPW1PW91 functional, we could examine the charge transfer efficiency between fragments of an electron D and A. The RE, which quantifies the potential for the transfer of charge from D to A parts, and is related to the electron and hole charge mobility, is a critical factor in the research and development of adequate materials for OSCs. According to Marcus’ theory, there is an inverse connection between RE and the movement of these charges (electrons and holes). As a result, the reduction in the value of the RE will cause the charge transfer to be completed with greater effectiveness.

The internal reorganization energy of organic solar cell molecules has a substantial impact on the device’s charge mobility, CT, and efficiency. Higher reorganization energy can lead to slower charge transfer rates, lower charge mobility, and decreased device efficiency. On the other hand, lower reorganization energy can result in faster charge transfer rates, higher charge mobility, and increased device efficiency. The internal reorganization energy of the organic solar cell molecule can also affect the stability of the device. If the reorganization energy is too high, the device may be unstable and prone to degradation. Therefore, optimizing the internal RE of the organic solar cell molecule is necessary to optimize the device’s performance.

**Figure 9:**
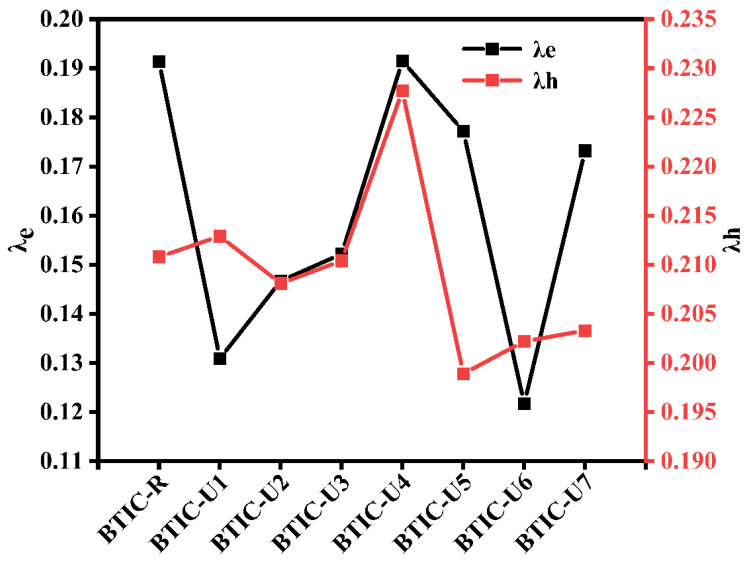
RE plot of λ_e_ and λ_h_ for BTIC−R and BTIC−U1 to BTIC−U7 molecules.

The RE is affected by various factors, including the geometry of cation and anion arrangements. The hole and electron exchange between acceptor and donor units is indicated by the cationic and anionic geometries, respectively. By solving Equations (6) and (7), we calculated the electron and hole RE values for each of the molecules under investigation; these values are summarized in Table 7 and Figure 9.

For BTIC-R molecule, the RE of λ_e_ and λ_h_ are 0.1914 eV and 0.2108 eV, respectively. Except for BTIC-U4, all of the modified molecules improve upon the mobility of electrons compared to the reference BTIC-R by having lower RE energies for electrons. Based on their λ_e_ values, the molecules can be arranged as follows: BTIC-U6 < BTIC-U1 < BTIC-U2 < BTIC-U3 < BTIC-U7 < BTIC-U5 < BTIC-R < BTIC-U4.

Based on these calculations, all other hypothetical molecules have a smaller value of λ_e_ than BTIC-U4. In addition, BTIC-U6 conjugation framework and strong elctron withdrawing group in acceptor regions give it a minimum value of λ_e_ (0.1217 eV), meaning it has a better rate of electron flow and can perform extraordinarily well in the process of charge transfer. Regarding acceptor molecules, the BTIC-U6 molecule has been shown to be the most effective

Molecules with lower λ_h_ such asBTIC-U2, BTIC-U3, BTIC-U5, BTIC-U6, and BTIC-U7, compared to BTIC-R, have a greater hole mobility than BTIC-R. In order of increasing hole moblility, molecules can be arrangedas follows: BTIC-U5 < BTIC-U6 < BTIC-U7 < BTIC-U2 < BTIC-U3 < BTIC-R < BTIC-U1 < BTIC-U4. To maximize hole mobility, the end-group acceptor effectively contributes to reducing the hole RE of the BTIC-U5 and BTIC-U6 molecules. In light of these findings, it is clear that the mobility of holes formed by exciton dissociations is maximized in BTIC-U5 and BTIC-U6. But it’s easy to see that every molecule possesses λ_e_ < λ_h_. It demonstrates that electrons in these molecules are more likely to be mobile and generate current than holes are. All compounds’ lower λ_e_ values relative to their λ_h_ values suggest that they have improved acceptor capacities in modern organic photovoltaic cells. In addition, the low values of λ_e_ and λ_h_ for BRIC-U6 suggest that it may be the most stable molecule. In a nutshell, the improved performance of the newly proposed molecules in terms of internal reorganization energy can be interpreted to apply to the vast majority of them. As a result, they hold greater promise for enhancing charge mobility and enhancing performance.

### 2.10. Chemical Reactivity Parameters

**Figure 10:**
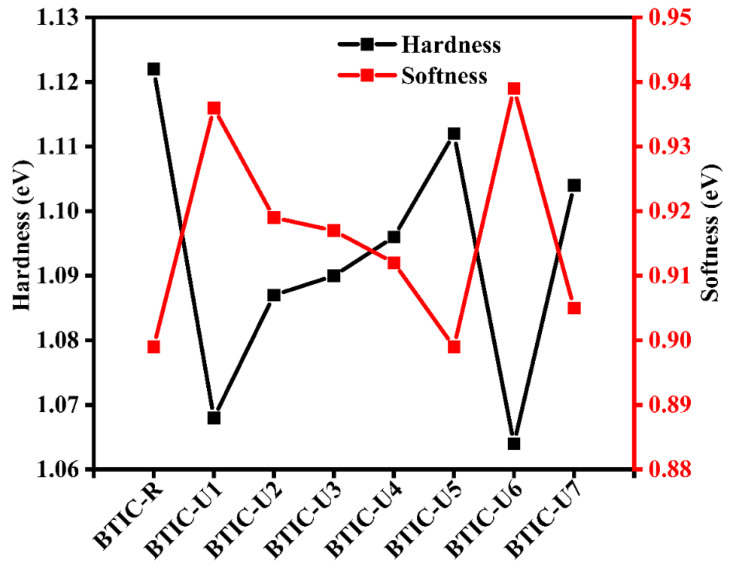
Hardness and softness of all the reference and newly designed molecules.

Chemical potential (μ), chemical hardness (η), chemical softness (S), and electrophilicity (ω) are all studied under the umbrella term “chemical-reactivity” to examine and compare the chemical behavior of BTIC-R and proposed molecules (BTIC-U1 to BTIC-U7). The term “electrophilicity” was coined to describe the phenomenon of an increased rate of electron flow in response to a decreased amount of energy. Table 8 shows that the values of all molecules, except for BTIC-U5, are higher than they were for BTIC-R, resulting in a good saturation of the designed structures with electrons. Compared to BTIC-R, the designed molecules have higher electrophilicity values, indicating that they function as acceptors. The chemical potential allows an acceptor to gain a net negative charge. To quantify the propensity of electrons to jump nuclei, the μ value is calculated for molecules with the BTIC-R and BTIC-U1 to BTIC-U7 hybrid electronic structure. The more significant negative potential of molecules in the range of BTIC-U1 to BTIC-U7 results in a marked increase in their reactivity. Chemical hardness, denoted by the formula η = 1/S, measures how well a material resists breakdown when subjected to chemical or physical stress. When an electron is added to the proposed structures, Table 8 below shows how much energy is lost (ensuring a negative chemical potential and keeping the hardness positive). The ability of the structures to transfer charge is explained by their pliability. The resistance to electron allocation decreases as S increases, so a material with a higher S value will have a softer surface (as shown in Figure 10).

### 2.11. Device Performance

The open circuit voltage (Voc) of any solar instrument can be measured to determine the instrument’s photovoltaic activity, which is an essential step in deciding how the mechanism operates. When no other load is applied to an optical device, the Voc value represents the complete voltage range made available by the device. The voltage output of a photovoltaic cell is affected by different factors, including the temperature of the solar device, charge transfer, and incident light, amongst others. The valence band of the donor material is typically coupled with the conduction band of the acceptor material, which results in optimal voltage. In order to achieve the desired increased Voc values, the donor compound must have a lesser HOMO level, while the acceptor unit must have an upper LUMO level.

To achieve a high PCE, a photovoltaic system requires a high fill factor, which rises as Voc rises. Voc and intermolecular energy gaps are inextricably linked for predicting the efficiency of a solar cell. Donor polymer PTB7-Th’s HOMO level is blended with BTIC-R LUMO level, and small acceptor molecules were claimed in this investigation, enhancing Voc (BTIC-U1 to BTIC-U7). PTB7-Th has a HOMO energy of −5.01 eV and a LUMO energy of −3.60 eV, suggesting it is a solid donor. In this study, Voc was determined statistically using Equation (2).

(2) $$Voc=\frac{E_{\mathrm{acceptor}\;}^{LUMO}-E_{\mathrm{donor}\;}^{HOMO}}{e}-0.3$$

**Figure 11:**
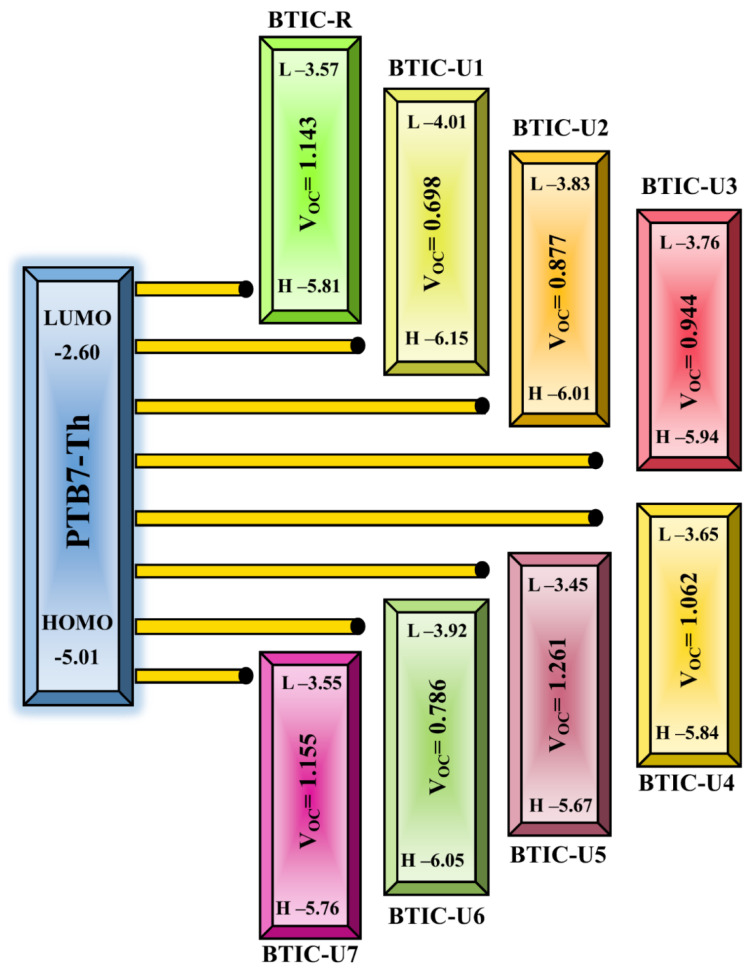
Voc of BTIC−R and BTIC−U1 to BTIC−U7 with respect to donor PTB7−Th polymer.

In the equation, *e* stands for the molecular charge, which has a value of 1, whereas the inter-surface charge is a separate factor that typically has a value of 0.3. It was discovered that the Voc of BTIC-R is 1.143 eV, and the range of Voc values for BTIC-U1 to BTIC-U7 molecules was found to be 0.698–1.261 eV. When contrasted with the BTIC-R molecule, the BTIC-U5 and BTIC-U7 molecules have a Voc higher than the BTIC-R molecule. The theoretically calculated Voc levels for all of the compounds studied relative to PTB7-Th are depicted in Figure 11, whereas the statistically calculated data are provided in Table 9.

The order of the molecules BTIC-R and BTIC-U1 to BTIC-U7 increased as follows: BTIC-U1 > BTIC-U6 > BTIC-U2 > BTIC-U3 > BTIC-U4 > BTIC-R > BTIC-U7 > BTIC-U5 According to this investigation, the BTIC-U5 molecule has the highest Voc value; as a result, it has the potential to be utilized in an effort to improve the PCE of OSCs.

To calculate the PCE of a PV system, one of the most crucial parameters is the fill factor (FF), which is directly related to the PCE. The Voc present at the interface between the acceptor molecule and the donor molecule significantly influences this aspect. To compute the FF of all of the molecules that we looked at, we used the equation shown 5 below.

(3) $$FF=\frac{\frac{eVoc}{K_{B}T}-\ln\left(\frac{eVoc}{K_{B}T}+0.72\right)}{\frac{eVoc}{K_{B}T}+1}$$

The normalized Voc is $\frac{eVoc}{K_{B}T}$, where the standard charge (*e*) is always 1, *K*_B_ stands for the Boltzmann constant, and the letter *T* stands for a constant temperature of 300 K. The Voc values for the compounds BTIC-R and BTIC-U1 to BTIC-U7 that were computed using computational methods are listed in Table 9 along with the normalized Voc and FF. The BTIC-R molecule has a normalized Voc of 44.146 and an FF of 0.893. The normalized Voc (48.674 and 44.597) and FF (0.901 and 0.894) of the BTIC-U5 and BTIC-U7 molecules are higher than those of the BTIC-R molecule due to their more excellent Voc. The results of the study show that these small molecules have amazing photoelectronic properties that may be useful in real-world applications.

The PCE, which sums up all of a solar cell’s performance metrics, can determine whether a photovoltaic material is practical enough to use. A molecule’s PCE is directly affected by the short circuit voltage (*Jsc*), the FF, and Voc. Whereas the strength of the radiation absorbed by the cell has the contrary effect. Equation (4) effectively describes this relationship.

(4) $$PCE=\frac{JscVocFF}{P_{in}}$$

By comparing each designed molecule to a standard reference molecule, we can see their Voc and FF values. It has been found that as Voc rises, so do FF and PCE. As can be seen in Table 9, BTIC-U5 and BTIC-U7 have the highest PCE and can be relied upon to improve the efficiency of solar devices.

### 2.12. Transition Density Matrix (TDM) and Binding Energy (Eb)

**Figure 12:**
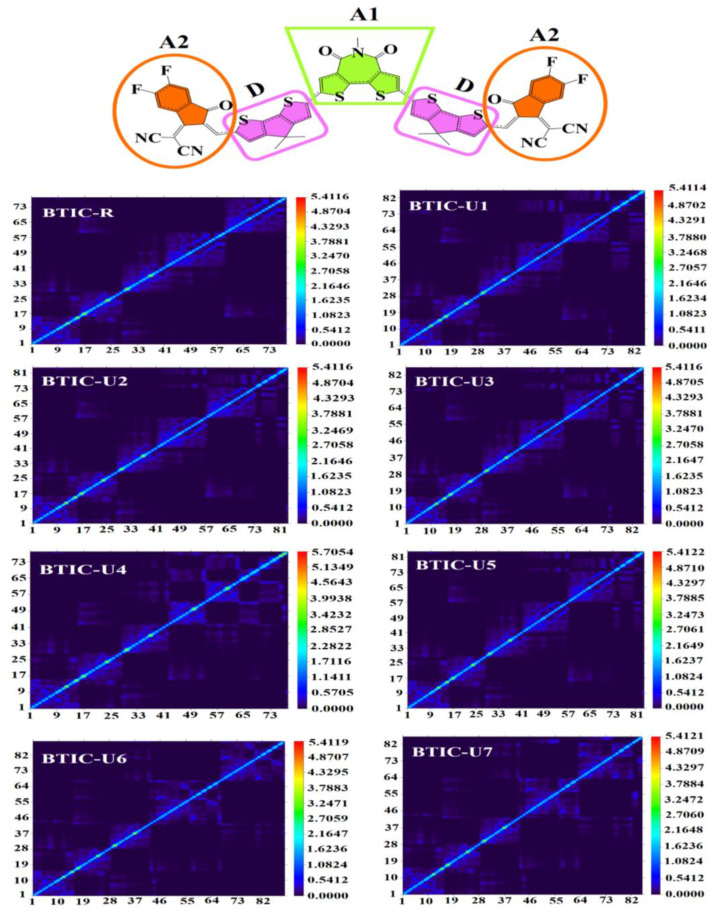
TDM plot of BTIC-R and BTIC-U1 to BTIC-U7 molecules.

In conjugated chemical systems, TDM analysis seems to be needed to figure out where exciton (hole electron pair) transition will happen amongst the D-A regions. This analysis could look at exciton mobility during the absorption and emission process in the excited state, fundamental charge locations, and charge transition prediction. TDM plots are generally utilized to evaluate electronic properties, such as the level of delocalization, and the effects of resonance, and to gain insight into the charge mobility inside a molecule. Due to its negligible charge transfer properties, hydrogen’s contribution to the transition is typically disregarded in TDM analysis. All atoms, with the exception of hydrogen, are represented by numbers on the left y- and bottom x-axes of the matrix, and the charge density coefficient is shown by the colored bars that progress from blue to red along the right y-axis. In order to determine the transition pathway, the molecule was split into three sections, which were labeled donor (D), acceptor (A), and acceptor1 (A1) in Figure 12. The charge density is found to be efficiently distributed in both the off-diagonal and diagonal directions throughout the molecules of all the compounds, with the diagonal direction responsible for the mainstream charge distribution. It was discovered that the flow of charge was effectively moving from donor to acceptor and acceptor1, which suggests that the whole molecule is enduring sequential conjugation.

The *E*_b_ of the excitons is added aspect that should be considered in this context. By measuring the binding energy, the cell’s operational efficiency, soar cell potential for hole-electron(exciton) dissociation, and its electrical properties can be estimated. The coulombic force between the hole and electron, denoted by *E*_b_, is the determining factor in their interactions. Less coulombic interaction exists between electrons and holes when *E*_b_ is low and vice versa. The *E*_b_ values in Table 10 were determined using the following Equation (5).

(5) $$E_{b}=E_{\mathrm{gap}\;}-E_{x}$$

*E*_x_ in the above equation is the solvent phase’s first excitation energy, and *E*_gap_ is the HOMO-LUMO gap. In the chloroform solvent, the BTIC-U1, BTIC-U4, and BTIC-U5 contain higher *E*_b_ than BTIC-R, whereas the BTIC-U2, BTIC-U3, BTIC-U6, and BTICU7 have lower *E*_b_ in comparison to the BTIC-R molecules. It is important to point out that the *E*_b_ of each molecule is comparable to the molecule that serves as the reference. Excitons present in molecules with a lower *E*_b_ value are able to easily diffuse into free charge carriers, enabling them a good option for boosting the charge density of an electric current. The device’s efficiency will increase as these (electron and hole) charge carriers move more quickly to their corresponding electrodes and produce more current. So, the BTIC-U6 molecule appears to have the lowest *E*_b_ among all molecules, making it the most competent candidate.

### 2.13. Charge Transfer Analysis

**Figure 13:**
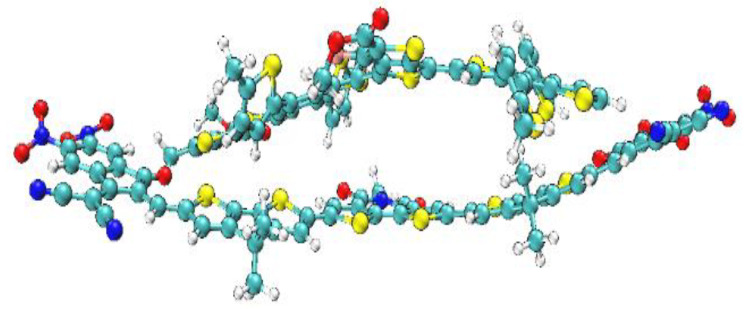
Optimized structure of PTb7-Th with BTIC-U1.

The investigation of the interaction between BTIC-U1 and the well-known donor-type polymer PTB7-Th is being done so that we can evaluate the charge transfer (CT) capabilities of the molecules that we have designed. We have completed an in-depth analysis of the complex depicted in Figure 13.

**Figure 14:**
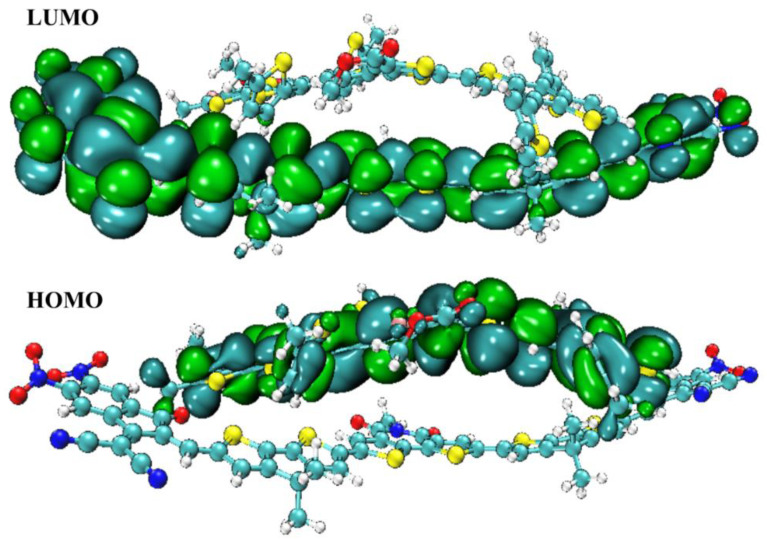
HOMO and LUMO distribution on the blend.

Significant dipole moment, subtle reorganization energy, low excitation energy, and sizeable bathochromic shift led us to choose BTIC-U1 over all other fabricated molecules. As a result, the BTIC-U1 molecule is selected as the best acceptor molecule. PTB7-Th, on the other hand, is a commonly used donor polymer in CT analysis. At the MPW1PW91/6-31G* functional, the composite made up of PTB7-Th and BTIC-U1 is optimized. Figure 14 depicts the BTIC-U1: PTB7-Th complex HOMO and LUMO pictorially. The figure clearly demonstrates that the donor polymer is aligned in a precisely parallel direction to the acceptor BTIC-U1. When undergoing CT or excitation, this orientation is optimal. The findings suggest that the density of charge in the HOMO state is located on the electron-rich donor moiety.

In contrast, the density of electrons in the LUMO state is distributed across the acceptor molecule of BTIC-U1. This distribution proved that electrons were transferred from the donor molecule to the acceptor atom. This change, which shows the superposition of orbitals between molecules, is a crucial confirmation of charge transfer between different moieties. Therefore, this acceptor group has been shown to be helpful for the effective molecular design of organic solar devices.

## 3. Computational Methodology

The latest method for studying quantum mechanics in computing is called density functional theory, or DFT. Due to this, all molecules’ geometric calculations were carried out using Gaussian 09 [52], and all three-dimensional molecular assemblies were created and viewed using GaussView 5.0 [53]. First, the reference structure (BTIC-R) [38] was optimized using the 6-31G* basis set and four exchange-correlation functionals: MPW1PW91 [54], CAM-B3LYP [55], B3LYP [56], ωB97XD [57], and PBEPBE [58]. The most promising of these functionals was then chosen after careful consideration, and subsequent analysis was performed on that one. TD-SCF simulations in solvent (chloroform) state was used to select the functional by comparing the resulting absorption spectra to the experimental spectra of BTIC-R [38]. Based on a comparison of the computed and experimental values of *λ_max_*, we concluded that the Modified Perdew Wang 1parameter (MPW1PW91) functional [54] is most coherent with the latter, thus providing a solid indication that this functional and the basis set will calculate modified molecules’ photovoltaic properties.

At this point, the absorption maximum results were processed using GaussSum [59], and the spectral representation was displayed using the Origin 2022 program. The density of states (DOS) and transition density matrix data of the BTIC-R and BTIC-U1 to BTIC-U7 molecules were analyzed, and the roles of various fragments of the molecules were determined with the help of Multiwfn 3.7 [60], PyMOlyze 2.0 [59] softwares.

The Marcus theory can be used to analyze the reorganization energy (RE), an essential factor for determining the number of intramolecular or intermolecular charge movements. Our investigations, however, are primarily concerned with intramolecular charge transfer (ICT). The overall RE comprises both the internal and external RE. Likewise, instances of external RE include polarization changes during charge transfer and abrupt changes in the outer atmosphere. Variability in the structures of molecules is involved in the internal RE. Environmental conditions affect external RE, making it difficult to calculate [61]. In this particular study, we concentrated solely on RE performed internally. We measured the anions ($\lambda_{-}$), and cations ($\lambda_{+}$) mobility, which together makes up the internal RE.

(6) $$\lambda_{+}=\left[E_{+}^{0}-E_{0}\right]+\left[E_{0}^{+}-E_{+}\right]$$

(7) $$\lambda_{-}=\left[E_{-}^{0}-E_{0}\right]+\left[E_{0}^{-}-E_{-}\right]$$

Optimized neutral molecular geometries yield cation and anion energies $E_{0}^{+}$ and $E_{0}^{-}$. $E_{+}$ and $E_{-}$ are optimized cation and anion geometries. Neutral molecule energies $E_{-}^{0}$ and $E_{+}^{0}$ are calculated using optimal anion and cation structures. $E_{0}$ is the optimised neutral molecule’s ground state point charge [62].

## 4. Conclusions

Seven new small molecule acceptors (BTIC-U1 to BTIC-U7) of the A2-D-A1-D-A2 type were theoretically developed in the current study to increase the efficiency of organic solar cells by changing the end-group of the reference (BTIC-R) molecule. Reduction in the energy band gap of up to 0.12 eV lower than BTIC-R (2.24 eV) is observed in designed molecules BTIC-U1 to BTIC-U7 with band gap values in the ambient of 2.12–2.22 eV. The DFT computed *λ_max_* value of 713 nm of the reference molecule shows good agreement with the experimental *λ_max_* value of 696 nm. The designed molecules are near-infrared sensitive, and showed a red shift in the absorption spectrum, and are broader—in the range of 680–707 nm (gas) and 723–761nm (chloroform)—than the phase values of BTIC-R, with lower transition energy values of 670 nm (gas) and 713 nm (chloroform). The RE of BTIC-R molecule for electron and hole were 0.1914 eV and 0.2108 eV, respectively, and the range of RE for electron and hole of freshly proposed molecules was seen to be 0.1217–0.1915 eV and 0.1989–0.2277 eV, correspondingly. The open circuit voltages (Voc) and PCE of BTIC-U1 to BTIC-U7 with respect to HOMO_PBTB-Th_–LUMO_acceptor_ are found with efficient values. LHE of BTIC-U1 to BTIC-U7 molecules had values ranging from 0.999224 to 0.999885. Our findings suggest that a straightforward and efficient alternative approach for creating promising NFAs molecules is to add more end-capped electron-accepting units. This study demonstrates the superiority of the newly created molecules, recommending their use in creating high-performance organic solar cell devices in the future.

## Figures and Tables

**Table 1:** Calculated energy gap (*E*_gap_) and FMOs (HOMO, LUMO) energy of BTIC-R and BTIC-U1 to BTIC-U7.

Compounds	E_HOMO_ (eV)	E_LUMO_ (eV)	Energy Gap (eV)
BTIC-R	−5.81	−3.57	2.24
BTIC-U1	−6.15	−4.01	2.14
BTIC-U2	−6.01	−3.83	2.17
BTIC-U3	−5.94	−3.76	2.18
BTIC-U4	−5.84	−3.65	2.19
BTIC-U5	−5.67	−3.45	2.22
BTIC-U6	−6.05	−3.92	2.12
BTIC-U7	−5.76	−3.55	2.21

**Table 2:** Gas phase computed transition character, oscillator strength (ƒ), transition energy (eV), maximum wavelength (*λ_max_*) of BTIC-R, and BTIC-U1 to BTIC-U7.

Molecules	Cal. *λ_max_* (nm)	Exp. *λ_max_* (nm)	Electronic Excitation Energy (eV)	Oscillator Strength (*f*)	Major Transition
BTIC-R	670	696	1.8499	3.3638	HOMO→LUMO (95%)
BTIC-U1	699	696	1.7727	3.2453	HOMO→LUMO (94%)
BTIC-U2	689	696	1.7975	3.3996	HOMO→LUMO (94%)
BTIC-U3	688	696	2.3108	3.4397	HOMO→LUMO (94%)
BTIC-U4	683	696	1.8131	3.2777	HOMO→LUMO (94%)
BTIC-U5	680	696	1.8230	3.6336	HOMO→LUMO (93%)
BTIC-U6	707	696	1.7535	3.7263	HOMO→LUMO (92%)
BTIC-U7	684	696	1.8126	3.645	HOMO→LUMO (93%)

**Table 3:** Solvent phase computed transition character, oscillator strength (ƒ), transition energy (eV), maximum wavelength (*λ_max_*) of BTIC-R, and BTIC-U1 to BTIC-U7.

Molecules	Cal. *λ_max_* (nm)	Exp. *λ_max_* (nm)	Electronic Excitation Energy (eV)	Oscillator Strength (*f*)	Major Transition
BTIC-R	713	696	1.7376	3.6158	HOMO→LUMO (93%)
BTIC-U1	761	696	1.6284	3.1103	HOMO→LUMO (90%)
BTIC-U2	742	696	1.6692	3.5172	HOMO→LUMO (92%)
BTIC-U3	740	696	1.6751	3.5707	HOMO→LUMO (92%)
BTIC-U4	737	696	1.6811	3.4724	HOMO→LUMO (92%)
BTIC-U5	723	696	1.7145	3.8991	HOMO→LUMO (93%)
BTIC-U6	755	696	1.6404	3.9393	HOMO→LUMO (92%)
BTIC-U7	727	696	1.7046	3.9032	HOMO→LUMO (93%)

**Table 4:** LHE of BTIC-R and BTIC-U1 to BTIC-U7 in the gas and chloroform solvent.

Molecules	LHE (Gas)	LHE (Solvent)
BTIC-R	0.999567	0.999758
BTIC-U1	0.999432	0.999224
BTIC-U2	0.999602	0.999696
BTIC-U3	0.999637	0.999731
BTIC-U4	0.999472	0.999663
BTIC-U5	0.999768	0.999874
BTIC-U6	0.999812	0.999885
BTIC-U7	0.999774	0.999875

**Table 5:** Dipole moment (μ) of BTIC-R and BTIC-U1 to BTIC-U7 molecules.

Molecules	μ (Gaseous Phase)	μ (Solvent Phase)
BTIC-R	1.159	0.899
BTIC-U1	5.582	7.164
BTIC-U2	2.433	2.170
BTIC-U3	1.532	0.866
BTIC-U4	2.479	2.772
BTIC-U5	5.132	5.602
BTIC-U6	5.552	7.307
BTIC-U7	2.351	2.394

**Table 6:** Acceptor2, donor, and acceptor1 fragments involvement in forming FMOs of BTIC-R and BTIC-U1 to BTIC-U7 molecules.

Molecules		Acceptor1 (%)	Acceptor2 (%)	Donor (%)
BTIC-R	HOMO	30.5	18.1	51.4
	LUMO	24.0	39.0	37.0
BTIC-U1	HOMO	31.8	19.1	49.1
	LUMO	15.5	52.3	32.2
BTIC-U2	HOMO	31.0	19.0	50.0
	LUMO	18.7	46.7	34.6
BTIC-U3	HOMO	30.9	19.0	50.1
	LUMO	19.2	45.8	34.9
BTIC-U4	HOMO	30.4	19.3	50.3
	LUMO	19.0	48.1	32.9
BTIC-U5	HOMO	29.1	19.5	51.4
	LUMO	24.6	39.0	36.4
BTIC-U6	HOMO	30.6	20.0	49.4
	LUMO	17.4	48.2	34.5
BTIC-U7	HOMO	29.5	19.5	51.0
	LUMO	23.0	40.8	36.2

**Table 7:** RE of λ_e_ and λ_h_ for BTIC-R and BTIC-U1 to BTIC-U7 molecule.

Molecules	λ_e_	λ_h_
BTIC−R	0.1914	0.2108
BTIC−U1	0.1309	0.2129
BTIC−U2	0.1467	0.2081
BTIC−U3	0.1522	0.2104
BTIC−U4	0.1915	0.2277
BTIC−U5	0.1772	0.1989
BTIC−U6	0.1217	0.2022
BTIC−U7	0.1732	0.2033

**Table 8:** Global reactivity parameters of BTIC-R and BTIC-U1 to BTIC-U7.

Molecules	η (eV)	S (eV^−1^)	μ (eV)	ω (eV)
BTIC-R	1.122	0.899	−4.689	9.794
BTIC-U1	1.068	0.936	−5.080	12.083
BTIC-U2	1.087	0.919	−4.920	11.132
BTIC-U3	1.090	0.917	−4.856	10.814
BTIC-U4	1.096	0.912	−4.744	10.262
BTIC-U5	1.112	0.899	−4.561	9.355
BTIC-U6	1.064	0.939	−4.988	11.689
BTIC-U7	1.104	0.905	−4.659	9.829

**Table 9:** The computed Voc, normalized Voc, FF, and PCE of BTIC-R and BTIC-U1 to BTIC-U7.

Molecules	Voc (eV)	Normalized Voc	FF	PCE
BTIC−R	1.143	44.146	0.893	20.94
BTIC−U1	0.698	26.936	0.845	12.09
BTIC−U2	0.877	33.860	0.869	15.63
BTIC−U3	0.944	36.465	0.877	16.98
BTIC−U4	1.062	41.025	0.887	19.33
BTIC−U5	1.261	48.674	0.901	23.29
BTIC−U6	0.786	30.351	0.858	13.83
BTIC−U7	1.155	44.597	0.894	21.18

**Table 10:** *E_gap_*, *Ex*, and *E_b_* (chloroform solvent) of BTIC-R and BTIC-U1 to BTIC-U7 molecules.

Molecules	*E_gap_* (eV)	*E_x_* (eV)	*E_b_* (eV) Solvent
BTIC-R	2.2449	1.7376	0.5073
BTIC-U1	2.1361	1.6284	0.5077
BTIC-U2	2.1747	1.6692	0.5055
BTIC-U3	2.1802	1.6751	0.5051
BTIC-U4	2.1929	1.6811	0.5118
BTIC-U5	2.224	1.7145	0.5095
BTIC-U6	2.1285	1.6404	0.4881
BTIC-U7	2.2088	1.7046	0.5042

## Data Availability

Not applicable.
